# Evaluation of Lipid Profile Management in Coronary Artery Disease Patients on Statin Therapy: A Single-Centre, Retrospective, Observational Study

**DOI:** 10.7759/cureus.71942

**Published:** 2024-10-20

**Authors:** C Sridevi, Susheel Malani, Prakash H Chaudhary, Digvijay D Nalawade, Ankit Shokeen

**Affiliations:** 1 Cardiology, Dr. D. Y. Patil Medical College, Hospital, and Research Centre, Dr. D. Y. Patil Vidyapeeth (Deemed to be University), Pune, IND; 2 Internal Medicine, Dr. D. Y. Patil Medical College, Hospital, and Research Centre, Dr. D. Y. Patil Vidyapeeth (Deemed to be University), Pune, IND

**Keywords:** cardiovascular disease, coronary artery disease, dyslipidemia, ldl-c, lipid profiles

## Abstract

Background: Lipid control is crucial in managing coronary artery disease (CAD) to reduce cardiovascular risk. This study aimed to evaluate the effectiveness of lipid-lowering therapy, particularly statins, in achieving target lipid levels in patients with CAD.

Materials and methods: This single-center, retrospective, observational study was conducted at the Cardiology Outpatient Department of Dr. D. Y. Patil Medical College, Hospital and Research Centre, Pune, Maharastra, India. A sample size of 72 patients was included in this study. Adult patients receiving treatment for acute coronary syndrome (ACS), chronic stable angina (CSA), or unstable angina (UA) were included. Exclusion criteria were comorbidities affecting clinical decisions, HIV-positive status, pregnancy, or breastfeeding. Data on lipid profiles, including low-density lipoprotein (LDL) cholesterol (LDL-C), were collected, with LDL-C calculated using the Friedewald equation or measured directly. The primary outcome was the proportion of patients achieving LDL-C targets as per the 2019 European Society of Cardiology (ESC)/European Atherosclerosis Society (EAS) guidelines. Statistical analysis was performed using chi-square tests, with significance set at p < 0.05.

Results: Of the enrolled patients, a substantial proportion did not achieve target LDL-C levels, with LDL-C remaining above the recommended thresholds in a significant number of patients. Despite ongoing statin therapy, lipid control was suboptimal in many cases, particularly among those with elevated LDL-C levels. A detailed analysis of lipid profiles and the achievement of target levels was done, highlighting areas where current treatment strategies may fall short.

Conclusion: The study reveals that a significant number of patients with CAD on statin therapy do not achieve optimal lipid control, particularly in LDL-C management. This underscores the need for more aggressive or tailored lipid-lowering strategies to reduce residual cardiovascular risk in this high-risk population. Further studies are needed to explore the factors contributing to suboptimal lipid control and to develop interventions that can enhance the effectiveness of current therapies.

## Introduction

Asian Indian patients have four times the risk of coronary artery disease (CAD) compared to Caucasian patients, six times the risk of Chinese patients, and 20 times the risk of Japanese patients [1]. The issue in India is the incomplete identification, management, and control of CAD risk factors. India has three times higher age-standardized estimates of disability-adjusted life-years (DALYs) lost from CAD than wealthy nations [2,3].

Dyslipidaemia is a significant independent risk factor for CAD, which in turn promotes the development of atherosclerosis and related cardiovascular events. It has also been closely linked to the pathophysiology of cardiovascular diseases (CVDs) [4]. Asian populations, particularly Indians, have been found to have atherogenic dyslipidemia, with lower rates of high blood cholesterol and greater rates of low HDL cholesterol and high triglycerides than non-Asian populations [4,5]. Treatment is hampered by the lack of population-specific normal ranges and guidelines for several risk variables, including low-density lipoprotein cholesterol (LDL-C). 

The observed decrease in mortality linked to CAD in patients receiving optimal medical care can be primarily attributed to improved management of cardiovascular risk factors, including quitting smoking, effectively treating systemic hypertension, managing diabetes, and managing dyslipidemia. Specifically, the risk of developing obstructive CAD and related adverse events is decreased when statin medication is used extensively to lower LDL-C levels [6-8]. When given equivalent doses, pharmacokinetic investigations indicate that Indians attain higher levels of circulating statins than the Caucasian population. According to a Singaporean study, when given a single 40 mg dose of rosuvastatin, Asian Indians attained 1.68 times the plasma levels of the drug compared to the Caucasian population [9].

Few studies have examined the safety and effectiveness of statins in the Indian population. Indian patients have been managing their dyslipidemia by the National Cholesterol Education Program Adult Treatment Panel III (NCEP ATP III) recommendation [10]. Indians can obtain a 50% reduction in LDL levels even with moderate dosage statins, according to the Indian Reduction in LDL-Cholesterol through Statins (IRIS) trial [11]. It has been observed that patients receiving greater-intensity statins have a higher risk of developing new-onset diabetes compared to those receiving moderate-intensity statins; the incidence was higher in those with metabolic syndrome [12]. Asian Indians have not participated in a statistically meaningful number of large-scale statin trials that use high-intensity statins such as the Stroke Prevention by Aggressive Reduction in Cholesterol Levels (SPARCL) trial [13].

## Materials and methods

This was a single-center, retrospective observational study conducted at the Cardiology Outpatient Department of Dr. D. Y. Patil Medical College, Hospital and Research Centre, Pune, Maharashtra, India, from August 1, 2023 to July 31, 2024, to evaluate lipid profiles on follow-up visits in patients with CAD who were receiving statin therapy. The study was approved by the Institutional Ethics Sub-Committee of Dr. D. Y. Patil Medical College, Hospital, and Research Centre (approval number: I.E.S.C./W/164/2024). The requirement for informed consent was waived due to the retrospective nature of the study.

The study included patients aged over 18 years who had been treated for acute coronary syndrome (ACS), chronic stable angina (CSA), or unstable angina (UA) through percutaneous coronary intervention (PCI) or medical therapy. Data were collected from medical records, focusing on patients on statins for at least six months before their follow-up visit. Patients with incomplete records, comorbidities that significantly influenced clinical decision-making, HIV-positive status, pregnancy, or those who were breastfeeding were excluded.

The lipid profiles, including low-density lipoprotein (LDL) cholesterol (LDL-C) levels, were analyzed using data from previous visits, with LDL-C calculated using the Friedewald equation or directly measured if triglyceride levels exceeded 400 mg/dL. The primary outcome was the proportion of patients who achieved LDL-C targets according to the 2019 European Society of Cardiology (ESC)/European Atherosclerosis Society (EAS) guidelines.

A sample size of 72 patients was included in this study, providing adequate power to detect differences in LDL-C target achievement between the study population and previous reports. A post hoc power analysis demonstrated that the study had a power of 99.99% to detect a significant difference between the proportion of patients who did not achieve target LDL-C levels in our study (75.0%) compared to the 64.2% reported by Agarwal et al. [14] (p < 0.05). This high statistical power indicates that the sample size was more than sufficient to reliably assess the primary outcomes.

The 72 patients included in this study were selected through a consecutive sampling method. All patients who met the inclusion and exclusion criteria during the study period were considered eligible. This consecutive sampling ensured that the study captured a representative cohort from the clinical practice during the defined study period, without introducing selection bias. Therefore, the final sample of 72 patients represents all eligible and available cases during the designated time frame.

Statistical analysis was conducted using IBM SPSS Statistics for Windows, Version 26.0 (Released 2019; IBM Corp., Armonk, United States). Differences between proportions were tested for statistical significance using the chi-square test, with a p-value of < 0.05 considered statistically significant.

## Results

The age distribution reveals that the majority of patients were aged 46-60 years (43.1%) and above 60 years (43.1%), with a male predominance (66.7%). Diagnostically, the most common condition was ST-elevation myocardial infarction (STEMI) (59.7%), followed by non-STEMI (NSTEMI) (22.2%) and CSA (18.1%). Treatment interventions varied, with 19.4% undergoing coronary angiography (CAG) and 2.8% requiring coronary artery bypass grafting (CABG), while 11.1% were managed medically. Comorbidities included hypertension (50.0%), diabetes mellitus (34.7%), and a smaller proportion of patients had a history of smoking (6.9%) or obesity (1.4%) (Table 1).

**Table 1:** Distribution of study participants based on demographic, clinical, and treatment profiles NSTEMI: non-ST-elevation myocardial infarction; STEMI: ST-elevation myocardial infarction; ACE: angiotensin-converting enzyme; ARB: angiotensin II receptor blocker; AS: atorvastatin; RS: rosuvastatin

Variable	Category	Frequency	Percentage
Age (in years)	29-45	10	13.9%
	46-60	31	43.1%
	>60	31	43.1%
Sex	Female	24	33.3%
	Male	48	66.7%
Diagnosis	Chronic Stable Angina	13	18.1%
	NSTEMI	16	22.2%
	STEMI	43	59.7%
Coronary Angiography		14	19.4%
Coronary Artery Bypass Grafting		2	2.8%
Medical Management		8	11.1%
Diabetes Mellitus		25	34.7%
Hypertension		36	50.0%
Smoking		5	6.9%
Obesity		1	1.4%
ACE Inhibitor/ARB	Olmesartan 40 mg	2	2.8%
	Ramipril 2.5 mg	22	30.6%
	Ramipril 5 mg	5	6.9%
	Telmisartan 20 mg	3	4.2%
	Telmisartan 40 mg	21	29.2%
	Valsartan 50 mg	2	2.8%
Beta Blocker	Bisoprolol 2.5 mg	1	1.4%
	Carvedilol 3.125 mg	10	13.9%
	Metoprolol 12.5 mg	1	1.4%
	Metoprolol 25 mg	34	47.2%
	Metoprolol 50 mg	14	19.4%
Statin	AS 10 mg	6	8.3%
	AS 20 mg	9	12.5%
	AS 40 mg	24	33.3%
	RS 10 mg	10	13.9%
	RS 20 mg	16	22.2%
	RS 40 mg	7	9.7%

The mean total cholesterol was 141.4 ± 34.9 mg/dL with a median of 138 mg/dL, and 94.4% of patients had levels below 200 mg/dL. The mean triglyceride level was 130.6 ± 64.2 mg/dL, with 75.0% of patients having levels below 150 mg/dL. The mean high-density lipoprotein (HDL) cholesterol was 38.7 ± 11.5 mg/dL, with 59.7% of patients having levels below 40 mg/dL, indicating a higher cardiovascular risk. Non-HDL cholesterol had a mean of 102.7 ± 32.8 mg/dL, with nearly half (47.2%) of the patients having levels below 100 mg/dL. Lastly, the mean LDL-C was 78.1 ± 28.5 mg/dL, with 75.0% of patients having levels above the target of 55 mg/dL, highlighting the challenge in achieving optimal LDL-C control in this population (Table 2).

**Table 2:** Distribution of study patients based on lipid profile parameters HDL: high-density lipoprotein; LDL: low-density lipoprotein

Parameter	Mean	Median	SD	Range	Levels	n (%)	Reference range
Total Cholesterol (in mg/dL)	141.4	138	34.9	184.3	>200	4 (5.6)	<200
					<200	68 (94.4)	
Triglycerides (in mg/dL)	130.6	126.5	64.2	384	>150	18 (25.0)	<150
					<150	54 (75.0)	
HDL (in mg/dL)	38.7	36	11.5	55	<40	43 (59.7)	≥40 (Men), ≥50 (Women)
					>40	29 (40.3)	
Non-HDL (in mg/dL)	102.7	103.4	32.8	178	<100	34 (47.2)	<100
					>100	38 (28.8)	
LDL (in mg/dL)	78.1	76.4	28.5	139.5	>55	54 (75.0)	<55
					<55	18 (25.0)	

Analysis revealed that sex was significantly associated with non-HDL levels (p = 0.030). Specifically, 56.3% of male patients had non-HDL levels ≤100 mg/dL, while only 29.2% of female patients fell into this category. Age, diagnosis, CABG, diabetes mellitus, hypertension, smoking, and statin drug type did not show statistically significant associations with non-HDL cholesterol levels. The distribution across these variables was relatively balanced, with non-HDL cholesterol >100 mg/dL being more prevalent in certain subgroups, such as the female gender (70.8%) and those without coronary artery graft (56.9%) (Table 3).

**Table 3:** Association of non-HDL cholesterol levels with demographic and clinical characteristics * - Chi square test was applied. NSTEMI: non-ST-elevation myocardial infarction; STEMI: ST-elevation myocardial infarction; ACE: angiotensin-converting enzyme; ARB: angiotensin II receptor blocker; ASL atorvastatin; RS: rosuvastatin; HDL: high-density lipoprotein

Variable		Non-HDL level (mg/dL)		Total, n (%)	p-value*
		≤100, n (%)	>100, n (%)		
Age (in years)	29-45	6 (60.0)	4 (40.0)	10 (100)	0.684
	46-60	14 (45.2)	17 (54.8)	31 (100)	
	>60	14 (45.2)	17 (54.8)	31 (100)	
Sex	Male	27 (56.3)	21 (43.8)	48 (100)	0.030
	Female	7 (29.2)	17 (70.8)	24 (100)	
Diagnosis	Chronic Stable Angina	4 (30.8)	9 (69.2)	13 (100)	0.421
	NSTEMI	8 (50.0)	8 (50.0)	16 (100)	
	STEMI	22 (51.2)	21 (48.8)	43 (100)	
Coronary Angiography	Yes	9 (64.3)	5 (35.7)	14 (100)	0.154
	No	25 (43.1)	33 (56.9)	58 (100)	
Coronary Artery Bypass Grafting	Yes	0 (0.0)	2 (100.0)	2 (100)	0.175
	No	34 (48.6)	36 (51.4)	70 (100)	
Medical Management	Yes	5 (62.5)	3 (37.5)	8 (100)	0.359
	No	29 (45.3)	35 (54.7)	64 (100)	
Diabetes Mellitus	Yes	13 (52.0)	12 (48.0)	25 (100)	0.554
	No	21 (44.7)	26 (55.3)	47 (100)	
Hypertension	Yes	15 (41.7)	21 (58.3)	36 (100)	0.345
	No	19 (52.8)	17 (47.2)	36 (100)	
Smoking	Yes	3 (60.0)	2 (40.0)	5 (100)	0.553
	No	31 (46.3)	36 (53.7)	67 (100)	
Statin	Atorvastatin	19 (48.7)	20 (51.3)	39 (100)	0.782
	Rosuvastatin	15 (45.5)	18 (54.5)	33 (100)	

The majority of patients with higher LDL-C levels (>55 mg/dL) were aged between 29-45 years (90.0%), followed by those aged 46-60 years (67.7%) and over 60 years (77.4%). A higher percentage of female patients (83.3%) had LDL-C levels >55 mg/dL compared to male patients (70.8%), though this difference was not statistically significant (p=0.248). Regarding diagnosis, 92.3% of patients with CSA had LDL-C levels >55 mg/dL, compared to 75.0% of NSTEMI and 69.8% of STEMI patients. Although differences were observed, they were not statistically significant (p=0.259). For patients who underwent CABG, all had LDL-C levels >55 mg/dL, while patients managed medically had a slightly lower percentage (62.5%). The presence of comorbidities like diabetes mellitus (72.0%) and hypertension (80.6%) was associated with higher LDL-C levels; however, these findings were not statistically significant. Patients treated with atorvastatin were more likely to have LDL-C levels >55 mg/dL (79.5%) compared to those on rosuvastatin (69.7%), with no significant difference (p=0.339) (Table 4).

**Table 4:** Association of LDL cholesterol levels with demographic and clinical characteristics * - Chi square test was applied. NSTEMI: non-ST-elevation myocardial infarction; STEMI: ST-elevation myocardial infarction; ACE: angiotensin-converting enzyme; ARB: angiotensin II receptor blocker; AS: atorvastatin; RS: rosuvastatin; LDL: low-density lipoprotein

Variable		LDL level (in mg/dL)		Total, n (%)	p-value*
		>55, n (%)	≤55, n (%)		
Age (in years)	29-45	9 (90.0)	1 (10.0)	10 (100.0)	0.338
	46-60	21 (67.7)	10 (32.3)	31 (100.0)	
	>60	24 (77.4)	7 (22.6)	31 (100.0)	
Sex	Male	34 (70.8)	14 (29.2)	48 (100.0)	0.248
	Female	20 (83.3)	4 (16.7)	24 (100.0)	
Diagnosis	Chronic Stable Angina	12 (92.3)	1 (7.7)	13 (100.0)	0.259
	NSTEMI	12 (75.0)	4 (25.0)	16 (100.0)	
	STEMI	30 (69.8)	13 (30.2)	43 (100.0)	
Coronary Angiography	Yes	11 (78.6)	3 (21.4)	11 (78.6)	0.701
	No	43 (74.1)	15 (25.9)	43 (74.1)	
Coronary Artery Bypass Grafting	Yes	2 (100.0)	0 (0.0)	2 (100.0)	0.408
	No	52 (74.3)	18 (25.7)	70 (100.0)	
Medical Management	Yes	5 (62.5)	3 (37.5)	8 (100.0)	0.386
	No	49 (76.6)	15 (23.4)	64 (100.0)	
Diabetes Mellitus	Yes	18 (72.0)	7 (28.0)	25( 100.0)	0.668
	No	36 (76.6)	11 (23.4)	47 (100.0)	
Hypertension	Yes	29 (80.6)	7(19.4)	36 (100.0)	0.276
	No	25 (69.4)	11(30.6)	36 (100.0)	
Smoking	Yes	4(80.0)	1(20.0)	5(100.0)	0.789
	No	50(74.6)	17(25.4)	67(100.0)	
Statin	Atorvastatin	31(79.5)	8(20.5)	39(100.0)	0.339
	Rosuvastatin	23(69.7)	10(30.3)	33(100.0)	

## Discussion

The results of this study indicate that the patient cohort predominantly comprised older adults (ages 46-60 and >60), with a male predominance. STEMI was the most common diagnosis, and while a significant portion of patients underwent CAG, only a few required CABG, with others managed medically. The lipid profile analysis revealed that while most patients maintained total cholesterol and triglyceride levels within recommended ranges, HDL cholesterol levels were concerningly low in the majority, indicating elevated cardiovascular risk. Furthermore, despite the use of statins, a significant proportion of patients struggled to achieve target LDL levels, particularly among those with CSA and those treated with atorvastatin. The analysis also highlighted a significant association between sex and non-HDL cholesterol levels, with male patients more likely to have levels within the desired range compared to female patients. These findings underscore the challenges in managing lipid levels in this patient population, particularly in achieving optimal LDL and HDL targets, which are crucial for reducing cardiovascular risk. 

The Myocardial Ischemia Reduction With Immediate Cholesterol Lowering (MIRACL) trial was the first to show the advantages of starting statins right away following ACS [15]. A total of 3086 patients participated in the placebo-controlled randomized trial that began 24-96 hours after ACS, with treatment continuing for 16 weeks at a dose of 80 mg/day of atorvastatin. The composite primary outcome, which included nonfatal acute MI, resuscitated cardiac arrest, and recurring symptomatic myocardial ischemia, significantly decreased. The Pravastatin or Atorvastatin Evaluation and Infection Therapy-Thrombolysis in Myocardial Infarction 22 (PROVE IT-TIMI 22) trial, which compared two active comparators, was the next significant trial investigating the use of statins in ACS [16]. In the PROVE IT-TIMI 22 study, 4162 patients with ACS were randomized to receive either 40 mg/day of pravastatin or 80 mg/day of atorvastatin. The patients experienced an ACS event within 10 days before randomization.

The Incremental Decrease in End Points Through Aggressive Lipid Lowering (IDEAL) trial, which included 8888 patients and more than 20,000 patient-years of follow-up, confirmed the efficacy and safety of intensive statin therapy in the treatment of stable CAD [17]. All major societies' guidelines, which are based on this evidence, advise high-intensity statins for all patients with established CAD, particularly for those under 75 years of age [18]. Poor control of LDL-C was identified in almost 60% of individuals with cardiovascular disease, according to a Spanish study [19]. More than one-third of the patients in different research on type 2 diabetic patients conducted in Ghana had high levels of LDL and total cholesterol [20]. In a Mexican investigation, individuals with early CAD who underwent lipid control assessment using two distinct criteria were also found to have suboptimal lipid control [21]. Furthermore, investigations done in Spain [22] and India [23] revealed inadequate cholesterol management in congenital heart disease (CHD) patients.

The findings of our study align with those reported by Agarwal et al., who also observed substantial challenges in achieving optimal lipid profile control in patients with documented CAD despite ongoing statin therapy [14]. In their study, 64.2% of high-risk patients did not achieve target LDL-C levels, which is consistent with our finding that 75.0% of our cohort had LDL-C levels above the recommended threshold of 55 mg/dL. Both studies highlight the difficulty in managing LDL-C even with intensive lipid-lowering treatment, underscoring the need for more aggressive or tailored therapeutic strategies. Additionally, while Agarwal et al. reported abnormal HDL cholesterol levels in 35% of patients [14], our study found a higher prevalence of low HDL cholesterol, with 59.7% of patients falling below the recommended levels, further emphasizing the persistent challenge in managing HDL in CAD patients. The elevated triglyceride levels noted in 25% of Agarwal et al.'s patients [14] also mirror our findings, where a significant portion of patients had elevated triglycerides, despite adherence to therapy. These comparative results suggest that achieving comprehensive lipid control remains a significant hurdle in the secondary prevention of cardiovascular events, necessitating ongoing evaluation and potentially more aggressive or combination lipid-lowering strategies in clinical practice.

This study has several limitations that should be considered when interpreting the results. First, the single-center design may limit the generalizability of the findings to broader populations. Second, the cross-sectional nature of the study only provides a snapshot of lipid control at a single point in time, without accounting for longitudinal changes or adherence to therapy over time. Third, the study relied on medical records, which may have introduced inaccuracies in reported data.

## Conclusions

The present study highlights the persistent challenges in achieving optimal lipid control in patients with CAD despite the use of statin therapy. A significant proportion of patients, particularly those with elevated LDL and low HDL levels, did not reach the recommended lipid targets, highlighting a potential gap in current treatment strategies. The findings suggest that more aggressive or individualized approaches may be necessary to manage dyslipidemia effectively in this high-risk population. Continuous monitoring and possibly the addition of other lipid-lowering agents or lifestyle interventions may be crucial in reducing residual cardiovascular risk and improving long-term outcomes for CAD patients.
